# Child Temperament as a Moderator of Promoting First Relationships Intervention Effects Among Families in Early Head Start

**DOI:** 10.1007/s11121-022-01340-0

**Published:** 2022-01-21

**Authors:** Jason T. Hustedt, Alison Hooper, Rena A. Hallam, Jennifer A. Vu, Myae Han, Melissa Ziegler

**Affiliations:** 1 Department of Human Development and Family Sciences, University of Delaware, 111 Alison Hall West, Newark, DE 19716 USA; 2 University of Alabama, Tuscaloosa, USA; 3 University of Delaware, Newark, USA

**Keywords:** Parent–child interactions, Early childhood intervention, Temperament, Early Head Start, Parenting stress

## Abstract

As availability of parent–child interaction curricula increases, Early Head Start (EHS) provides a relevant context to test research-based parenting models as part of everyday practice. We trained EHS staff to incorporate the Promoting First Relationships (PFR) intervention into ongoing weekly home visits with mothers and their young children (*n* = 102) enrolled in EHS. Children had a mean age of 19.75 months and were 56% Hispanic, 23% Black, and 14% White. Families were randomly assigned to an intervention group where they participated in PFR as an EHS enhancement, or to a waitlist-control group where they received only typical EHS services. To explore the possibility that effectiveness of parent–child curricula may differ based on child characteristics, we used linear regression to examine children’s temperament as a potential moderator of PFR efficacy on outcomes related to parenting stress, family functioning, and parent–child interaction. While we did not find a significant main effect of PFR for the full sample, there were several significant moderated effects. For families where children showed higher levels of surgency, mothers’ parenting stress was significantly reduced after PFR participation. Also, when children showed higher levels of negative affect, mothers demonstrated higher sensitivity in parent–child interactions after participating in PFR. Given findings from our exploratory study, agencies should consider the characteristics of families served and the match with intervention priorities, when selecting intervention programs. When delivered as a home visitation enhancement, PFR may be a valuable support for certain enrolled families, based on child characteristics including high levels of surgency or negative affect.

**Supplementary Information:**

The online version contains supplementary material available at 10.1007/s11121-022-01340-0.

Positive interactions between parents and young children can help set the stage for positive outcomes later in children’s lives, and the parent–child relationship has been identified as a buffering mechanism against potential negative effects of risks such as poverty and high family stress (Buffering Toxic Stress Consortium Principal Investigators et al., 2013). There has been great interest in developing curricula or interventions seeking to promote positive parent–child interactions. Evidence-based early childhood interventions are increasingly being employed in real-world settings, now reaching communities and populations beyond those included in model studies used to develop the interventions. The extension of these interventions into new research contexts raises questions about ensuring their effectiveness under field conditions where child and family characteristics may vary more widely than in model studies. Our exploratory study examines the role played by child temperament in influencing the effectiveness of the Promoting First Relationships (PFR; Kelly et al., 2008a, b) intervention to promote positive family functioning. In our work as part of the Buffering Toxic Stress (BTS) Consortium, we tested the efficacy of PFR when offered by home visitors as an enhancement to typical Early Head Start services.

Early Head Start (EHS) is an ideal context to study parenting interventions in the field, given its emphasis on parent engagement and supporting families. EHS is a federally funded program for families with children ages 0–3 and pregnant mothers. It employs a two-generation approach, offering comprehensive services that address goals such as children’s social-emotional development and families’ self-sufficiency. Results from the multi-site randomized control Early Head Start Research and Evaluation Project (EHSREP; Love et al., 2005) show that EHS produces positive impacts for both parents (e.g., increases in language and emotional support in parent–child interaction) and children (e.g., less aggressive behavior, more emotional engagement). Though overall EHSREP effect sizes were modest, the researchers also found larger impacts when EHS programs tailored delivery of services based on family needs and when the EHS model was initially more fully implemented. While nearly all EHS families experience poverty and all experience elevated risk, variations in the way families perceive stress are in turn linked with differences in family functioning (Hustedt et al., 2017). As a result, some EHS families may benefit more than others from a parenting intervention. Also, within federal guidelines, EHS programs make decisions about specific services to offer their clients, including, potentially, parent–child interaction intervention models. Those decisions should be based on program characteristics as well as specific populations served; certain types of interventions may be a better match for certain EHS programs. Participation in well implemented EHS home visiting is linked with positive outcomes for parents as well as children (Jones Harden et al., 2012). One opportunity in EHS programs with home visitation models involves integrating delivery of parent–child interaction interventions into recurring home visits.

## Child Temperament and Parenting

In addition to studying the overall efficacy of parent–child intervention models, researchers have become interested in whether the interventions are equally effective across different populations of families. Individual differences on the part of both caregivers and young children are potentially important. Differential susceptibility perspectives highlight that some individuals may benefit more than others from the same enriching experience (Belsky & Pluess, 2009). Also, precision home visiting approaches in early childhood (Supplee & Duggan, 2019) are predicated upon the idea of matching home visiting strategies with the families most likely to benefit from those “ingredients.” In prior studies of parent–child interventions, the field has considered different characteristics of caregivers and how that might moderate outcomes of parenting interventions. However, study findings are mixed relative to the direction of such effects (Berlin et al., 2011). Child characteristics including temperament can also contribute to intervention efficacy. In BTS research on the Attachment and Biobehavioral Catch-up (ABC) intervention, Hepworth et al. (2020) found that emotional reactivity in infants moderated the effects of ABC on their problem behavior scores and emotion regulation outcomes, with more positive effects for more highly reactive infants. Moderated effects of temperament are of particular interest in the current study as well. In general, temperament refers to individual differences in children’s self-regulation and reactivity and broadly includes (1) surgency, or extraversion, which is related to positive emotionality, stimulation seeking, and impulsivity; (2) negative emotionality, which includes intense negative emotions, such as sadness, anger, and high irritability; and (3) effortful control, which refers to regulation of emotions and behaviors (Armour et al., 2018; Paulussen-Hoogeboom et al., 2007; Rothbart, 2011; Rothbart & Bates, 2006). While our focus is on temperament as a moderator, it is important to highlight not only that children’s temperaments are linked to parents’ temperaments, but also that other prior or concurrent experiences by children (e.g., neurodevelopmental differences and prenatal risks) and parents (e.g., anxiety, depression) can affect expression of young children’s temperament.

Much of the research examining child temperament and parenting focuses on children’s negative emotionality; however, findings are mixed. For instance, irritability in children has been associated with less parental sensitivity during child’s play, reduced parental responsiveness to children’s needs, and increased parental controlling and coercive behaviors (Armour et al., 2018; Bridgett et al., 2009; Ciciolla et al., 2013). Less research examines how surgency and effortful control affect parenting practices. Again, findings are mixed. While surgency is generally associated with less sensitive parenting, positive emotionality is associated with parenting that is less negative (Bridgett et al., 2013; Fields-Olivieri et al., 2017; Planalp et al., 2013). With regard to effortful control in young children, it is associated with less hostile, controlling, or overreactive parenting behaviors and higher quality parental responsiveness (Bridgett et al., 2009; Wilson & Durbin, 2012).

Research examining interactional effects often focuses on how differences in children’s levels of self-regulation and negative emotionality relate to their sensitivity to both parental control and responsiveness (Kiff et al., 2011). A meta-analysis by Slagt et al. (2016) found that while children with difficult temperaments experienced greater benefits of positive parenting, they were also generally more vulnerable if experiencing negative parenting. However, this relationship was only present when temperament was assessed during infancy. One study of parenting behavior and stress in EHS families found the highest levels of maternal negative regard among mother–child dyads composed of temperamentally reactive children and mothers experiencing high levels of stress (Dalimonte-Merckling & Brophy-Herb, 2019). Findings from studies such as these suggest that PFR and other early intervention curricula have potential to address temperament challenges for families in EHS by focusing on the parent–child relationship.

Moderating effects of temperament are also seen in distal child outcomes, particularly for children with more difficult temperaments. In a study of low-income families, child difficult temperament was a moderator of the relationship between maternal responsiveness and children’s externalizing problems (Kochanska & Kim, 2013). In a study by Stright et al. (2008), when infants experienced higher levels of parenting quality, those with difficult temperaments showed better adjustment in first grade than less difficult counterparts. If they had lower levels of supportive parenting, temperamentally difficult infants experienced poorer academic adjustment.

## Promoting First Relationships

Based in attachment theory, the PFR intervention is intended to support parents in addressing the social-emotional needs of infants and young children. PFR is manualized and consists of 10 sessions in which intervention service providers use specific consultation strategies (Kelly et al., 2008a, b) such as establishing a positive emotional climate for the parent and promoting parents’ capacities to be reflective when observing their child. Parents are encouraged to respond to children’s cues with empathy and understanding, use routines, and encourage exploration and play. Service providers also record short video segments of parents interacting with their children and then watch them with parents, reviewing PFR principles, highlighting strengths, and offering suggestions (Kelly et al., 2008a, b; Spieker et al., 2012). Results from a randomized trial by the PFR developers (Spieker et al., 2012) show that caregivers and toddlers involved in the child welfare system benefited from PFR, relative to control group participants. Caregivers showed more understanding of their toddlers and greater sensitivity, while toddlers showed higher levels of social competence. A second randomized trial (Oxford et al., 2016) was conducted with families referred to protective services for possible maltreatment, showing similar parenting benefits as well as reductions in child foster care placements. Using data from the same study, Pasalich et al. (2019) also found that positive parenting effects of PFR were moderated by parents’ prior experiences. Parents who had been physically (but not emotionally or sexually) abused showed more parental sensitivity than parents not abused as children.

## The Current Study

Our study is the first to examine the effectiveness of PFR as an enhancement to everyday EHS practice by home visitors. Beyond examining main effects of PFR, we were interested in exploring the role of child temperament as a possible moderator of PFR outcomes. Our overall research question was: Does child temperament influence the effectiveness of PFR in (1) improving family functioning, (2) reducing parenting stress, and (3) promoting positive parent–child interactions? We hypothesized that differences in child temperament would relate to differences in effectiveness of PFR. We further anticipated that all three dimensions of child temperament — negative affect, surgency, and effortful control — could be important, with some dimensions of temperament potentially emerging as most relevant. However, as this was an exploratory study, we did not make predictions about which dimensions of temperament would be related to measures of PFR effectiveness, or about directions of effect.

## Method

### Participants

Approximately 240 families from an EHS program and who met eligibility criteria were invited to participate. Families were enrolled on a rolling basis across the 3.5 years of the study. Families who consented (*n* = 190, 79%) were randomly assigned to either the PFR intervention group (*n* = 97) or to a waitlist-control group (*n* = 93). As our goal is to investigate moderated intervention effects, the intervention group in our analytic sample consists of families initially randomized to receive PFR and who completed the intervention. A total 102 families are included in the analytic sample, with 41 families in the PFR intervention condition and 61 in the control condition. Families excluded from the analytic sample include those who did not complete the intervention, dropped prior to post data collection, had missing data on all temperament constructs, or had incomplete data on demographic covariates. The control group includes families initially randomized to the control condition, regardless of whether they later completed PFR due to the waitlist-control design. This is not a no-treatment control, but rather a control group of families already receiving an early childhood intervention, EHS. The EHS program in this study further uses the Parents as Teachers (PAT) curriculum as part of its regular practice. Thus, the PFR families received an additional intervention layered atop those usual evidence-based early childhood intervention services, and the control group did not.

As shown in Table 1, children were primarily Hispanic and Black, non-Hispanic, with monthly incomes well below poverty thresholds. Most families (67.6%) received home-based EHS services. There were no significant differences between the intervention and control groups with respect to demographic variables. Families who were excluded from the analysis did not significantly differ from those in the analytic sample. A CONSORT diagram showing the flow of participants through the study is included in the Supplemental Materials.

**Table 1:** Demographic characteristics by group at baseline

	Full Sample (*n* = 102)	Control (*n* = 61)	Intervention (*n* = 41)	*p*
Child female	45.10%	47.14%	41.46%	0.545
Child race/ethnicity				0.748
White, non-Hispanic	13.73%	17.07%	11.48%	
Black, non-Hispanic	22.55%	19.51%	24.59%	
Hispanic	55.88%	53.66%	57.38%	
Multiracial or other race	7.84%	9.76%	6.56%	
Child age in months	19.75 (8.16)	20.14 (8.22)	19.18 (8.13)	0.564
Household monthly income	$1695.16 ($1068.56)	$1739.10 ($1018.89)	$1638.50 ($1140.76)	0.670
Caregiver highest level of education				0.905
Less than HS diploma	34 (33.33%)	21 (34.43%)	13 (31.71%)	
HS diploma or GED	25 (24.51%)	14 (22.95%)	11 (26.83%)	
Some college credits or higher	42 (41.18%)	25 (40.98%)	17 (41.46%)	
Child Temperament				
Effortful control	5.13 (0.91)	5.15 (0.89)	5.11 (0.94)	0.806
Negative affect	3.18 (1.13)	3.14 (1.10)	3.24 (1.20)	0.685
Surgency	5.17 (1.01)	5.26 (0.96)	5.05 (1.07)	0.331

### Procedure

Data collection protocols were administered in English or Spanish by research assistants, and Spanish-language versions of all written measures were employed as needed. After providing pre-test data, families assigned to PFR began the intervention as soon as possible. The PFR curriculum was delivered by EHS staff members (*n* = 17) who received extensive training in PFR from the intervention developers and a local Mentor/Coach. While another approach would be to hire new staff to focus only on intervention delivery (e.g., Berlin et al., 2018), we chose to train existing staff, to promote sustainability of PFR after our study ended. For families enrolled in home-based EHS, the interventionist was typically the family’s usual home visitor, and the PFR enhancement occurred during the family’s weekly home visit. For families in center-based EHS, this staff member was typically the family service worker assigned to their center, and home visits were added. Comparable versions of PFR materials are available in Spanish from the curriculum developers, and PFR was delivered in the family’s home language — English or Spanish. Follow-up post-data were collected once the family had completed PFR.

Families assigned to the control condition participated in pre- and posttest data collection sessions spaced about 14 weeks apart, based on the amount of time needed to complete PFR in pilot testing. During that time, the family continued to participate in “business as usual” by receiving all ongoing EHS services, including PAT, but not the PFR enhancement. The same measures were used for both pre- and post-test assessments, completed across two sessions at each time point. After initially attaining fidelity with PFR developers, home visitors and family service workers participated in annual fidelity checks with the developers, following the developers’ recommendations for PFR research. We employed a local EHS Mentor/Coach, certified by PFR developers, who provided ongoing resources, including additional fidelity checks. Only families who received PFR from a home visitor or family service worker currently maintaining fidelity were included in this study.

### Measures

Many of the measures used in the PFR intervention study were the same as those from a prior validation study (Hustedt et al., 2017) investigating family stress in our partner EHS program. Measures were selected based on age-appropriateness, availability in both English and Spanish, and prior use with low-income and ethnic minority families. A family demographics measure included items developed by the BTS Consortium (Buffering Toxic Stress Consortium Principal Investigators et al., 2013), as well as items developed for this study.

#### Parenting Stress

The Parenting Stress Index – Short Form (PSI-SF; Abidin, 1990) was used to measure parenting stress. The PSI-SF is a questionnaire containing 36 items, rated on a five-point scale (1, *Strongly Disagree*, to 5, *Strongly Agree*). It results in a Total Stress score and three subscale scores: Difficult Child, Parent–Child Dysfunctional Interaction, and Parental Distress. Higher scores indicate higher levels of stress. This measure has been widely used in research and can be successfully used as an indicator of stress in low-income families (Roggman et al., 1994). Subscales demonstrate good internal consistency (*α* = 0.80–0.87) and high test–retest reliabilities (*r* = 0.80–0.85) in previous research of families with infants and toddlers (Caley, 2012).

#### Family Functioning

The McMaster Family Assessment Device (FAD; Epstein et al., 1983) was used to measure family functioning. The FAD is a 60-item questionnaire that measures family functioning through seven inter-correlated scales: Problem Solving, Communication, Roles, Affective Responsiveness, Affective Involvement, Behavior Control, and General Functioning. Respondents rate their agreement with each item using a four-point scale, ranging from 1 (*Strongly Disagree*) to 4 (*Strongly Agree*). Higher scores indicate more unhealthy, or less optimal, family functioning. We used the 12-item General Functioning scale in this study. The General Functioning scale has been widely used in research, including studies of EHS families (e.g., McKelvey et al., 2015), and has been found to accurately measure family functioning on its own (Kabacoff et al., 1990). The scale has demonstrated adequate test–retest reliability in previous research (*r* = 0.71–0.77; Epstein et al., 1983; Evans et al., 2009).

#### Parent–Child Interactions

Parent–child interactions were assessed using a play-based direct assessment, the Three Bags Task. The Three Bags Task or a modification has been used in national studies of child development and parenting, including the NICHD Study of Early Child Care and Youth Development, the Family Life Project, and the Early Childhood Longitudinal Study–Birth Cohort (NICHD Early Child Care Research Network, 1997; Roisman & Fraley, 2008; Sulik et al., 2015). During this assessment, the child and a parent engage in a video-recorded, semi-structured 15-min play interaction, using three different sets of materials, beginning with the material in Bag 1 and ending with Bag 3. Materials included a wordless picture book, a puzzle-like toy, and an interactive toy.

Videos were coded by a team of trained coders at the University of North Carolina CDS Observes Center. Coders rated parents’ behavior on eight subscales: Sensitivity, Intrusiveness, Detachment, Stimulation for Development, Positive Regard, Negative Regard, Animation, and Dyadic Mutuality. Each subscale was rated using a 1–5 scale, where 1 represented *not at all*, and 5 represented *highly characteristic*. Coders were trained to initial reliability (ICC > 0.70) by a master coder. Ongoing inter-reliability checks were conducted for a minimum of 30% of videos (Cox & Crnic, 2002; Mills-Koonce, 2013).

Detachment and negative regard were reverse-scored before calculating composite scores. The coding team performed an exploratory factor analysis with oblique rotation using the eight subscale scores, which resulted in two composite scores that they provided to the research team: Positive Engagement, which consisted of Detachment, Positive Regard, Animation, and Stimulation for Development; and Sensitivity, which consisted of Sensitivity, Intrusiveness, and Negative Regard. A high score on Positive Engagement indicates that a parent was engaged and interested during play, showed affection, and attempted to foster learning. A high score on Sensitivity indicates that a parent was in tune with the child, respected the child’s efforts, and did not show signs of negative affect.

#### Child Temperament

Child temperament was measured using the Infant Behavior Questionnaire–Revised Very Short Form (IBQ-R VS; Putnam et al., 2014) and the Early Childhood Behavior Questionnaire Very Short Form (ECBQ VS; Putnam et al., 2010). Both measures are parent-report questionnaires that measure the emotional behavior of the child. The IBQ-R VS was used with children 3–12 months. The ECBQ VS was used with children 13–36 months. The IBQ-R VS has 37 items scored on a 1–7 Likert scale or as not applicable, and the ECBQ VS has 36 items, similarly scored. Both measures result in three subscales: surgency, negative affect, and effortful control. As the subscales show high continuity across the IBQ-R and ECBQ (Putnam et al., 2008), we combined scores for the IBQ-R VS and ECBQ VS.

### Analysis Strategy

Participant characteristics were summarized for outcome variables using independent samples t-tests to examine whether the control and intervention groups differed significantly at both pretest and posttest. Outcome measures included the three PSI subscales, the FAD General Functioning scale, and the two composite scores from the Three Bags Task. Next, to test the overall hypothesis of our exploratory study that effortful control, negative affect, and surgency dimensions of children’s temperament potentially moderated the effect of PFR on parent functioning, we fit a linear regression model to the data for a total of 18 models. Each model included adjusting covariates (child’s age, child’s sex, and child’s race and ethnicity), baseline score on the outcome measure, main effects for group (intervention or control), an ECBQ VS/IBQ-R VS subscale score, and the interaction between that subscale and group. Thus, 18 interactions were tested. The general moderator model is represented by the following equation:

$$\begin{aligned}\mathrm{Outcome}=& {\beta }_{0}+ {\beta }_{A}\mathrm{AGE}+{\beta }_{S}\mathrm{SEX}+{\beta }_{R}\mathrm{RACE}+{\beta }_{P}\mathrm{PRETEST}\\&+{\beta }_{G}\mathrm{GROUP}+{\beta }_{T}\mathrm{TEMPERAMENT}+{\beta }_{\mathrm{GT}}\mathrm{GROUP}\\&*\mathrm{TEMPERAMENT}\end{aligned}$$

Continuous variables were mean-centered before they were entered into the model. We included a random effect for home visitor or family service worker due to the nested nature of the data. Missing data were handled using listwise deletion. For each model, the model fit was assessed using graphical residual analysis. Tests for fixed effects were conducted using the Kenward-Roger approximation for the degrees of freedom, a bias-adjusted precision estimator, to account for the small sample size and unbalanced design. Overall model significance was tested using likelihood ratio chi-squared tests. Analyses were performed using SAS Version 9.4.

## Results

We examined differences between the control and intervention groups for the PSI, FAD, and Three Bags Task at baseline and follow-up data collection. See Table 2 for descriptive data for the full sample, control group, and intervention group, as well as results of significance tests. Intervention and control groups were comparable at baseline on outcome measures. There were no significant differences between groups at posttest. A correlation table in the Supplemental Materials shows relationships between outcome measures and temperament constructs.

**Table 2:** Descriptive statistics for outcome measures at pre/post by group

	Pre Test				Post Test			
	Full sample	Control	Intervention	*p*	Full sample	Control	Intervention	*p*
**PSI**								
Total Score	65.63 (21.56)	62.34 (19.49)	70.18 (23.68)	0.119	64.12 (22.01)	62.87 (22.48)	65.85 (21.56)	0.548
Parental Distress	25.35 (9.38)	24.44 (9.24)	26.56 (9.53)	0.290	24.25 (9.68)	23.92 (9.81)	24.69 (9.62)	0.709
Parent–Child Dysfunctional Interaction	17.71 (5.89)	16.63 (5.09)	19.16 (6.61)	0.053	17.60 (6.34)	17.29 (6.14)	18.00 (6.67)	0.611
Difficult Child	21.96 (8.57)	20.77 (8.12)	23.49 (9.00)	0.155	22.18 (8.74)	22.09 (9.21)	22.30 (8.23)	0.912
**Family Assessment Device**								
General Functioning	1.72 (0.52)	1.74 (0.52)	1.68 (0.51)	0.616	1.74 (0.47)	1.77 (0.48)	1.68 (0.46)	0.329
**Three Bags Test**								
Positive Engagement	3.43 (0.79)	3.44 (0.70)	3.41 (0.93)	0.890	3.41 (0.75)	3.39 (0.76)	3.44 (0.74)	0.749
Sensitivity	3.63 (0.79)	3.67 (0.79)	3.56 (0.79)	0.522	3.64 (0.73)	3.70 (0.76)	3.56 (0.68)	0.389

### Parenting Stress

To test our research question about temperament, for each of the three PSI subscales, we ran three models, one for each subscale of the ECBQ VS/IBQ-R VS as a moderator, for a total of nine models. Graphics depicting the interactions are included in the Supplemental Materials.

The overall model testing the interaction between surgency and group was significant (LRT: *χ*^2^_(df = 9)_ = 273.3, *p* < 0.001). There was a significant interaction between surgency and group membership (*F*_(1, 70)_ = 7.45, *p* = 0.008), which indicates that surgency moderated the effect of the PFR intervention on the Difficult Child score. Table 3 shows the full results for this model. The linear slope of the relationship between surgency and difficult child, adjusting for other covariates, was −3.24 for the intervention group and 0.93 for the control group. This means that for families in the intervention group, higher values of surgency were associated with lower Difficult Child scores; each one unit increase in surgency score was associated with a 3.24-unit reduction in the Difficult Child score.

**Table 3:** Regression models for PSI Difficult Child and Parent–Child Dysfunctional Interaction Subscales, Three Bags Test Sensitivity Composite, and Interactions

Variable	PSI Difficult Child			PSI Parent–Child Dysfunctional Interaction			Three Bags Sensitivity Composite		
	B	SE B	*p*	B	SE B	*p*	B	SE B	*p*
Pretest score (outcome variable)	0.68	0.09	< .001	0.72	0.10	< .001	0.55	0.08	<0.001
Surgency	0.93	1.23	0.149	0.61	0.94	0.076			
Negative affect							−0.19	0.07	0.259
Intervention group	−1.07	1.38	0.442	−0.99	1.11	0.376	−0.05	0.12	0.700
Interaction (surgency by group)	−4.18	1.53	0.008	−3.41	1.16	0.004			
Interaction (negative affect by group)							0.254	0.11	0.024
Child sex	−0.92	1.37	0.507	1.55	0.97	0.116	−0.06	0.12	0.624
Child race/ethnicity			0.396			0.274			<0.001
White, non-Hispanic	−0.82	2.28		0.04	1.76		0.029	0.20	
Hispanic	−2.15	1.63		−0.55	1.34		−0.02	0.16	
Multiracial or other race	−4.38	2.88		−3.93	2.13		−1.12	0.26	
Child age	0.26	0.10	0.009	0.03	0.08	0.734	0.01	0.01	0.184

For the model predicting PSI Difficult Child, *N* = 80. For the model predicting PSI Parent–Child Dysfunctional Interaction, *N* = 85. For the model predicting Three Bags Sensitivity Composite, *N* = 76. Only models where the interaction term was statistically significant are presented

For the Parent–Child Dysfunctional Interaction subscale of the PSI, the model testing the interaction between group and effortful control (*p* = 0.59) was not significant. The model testing the interaction between group and negative affect approached significance (*p* = 0.09). The overall model testing the interaction between surgency and group was significant (LRT: *χ*^2^_(df = 9)_ = 282.2, *p* < 0.001). There was a significant interaction between surgency and group membership (*F*_(1, 73.4)_ = 8.70, *p* = 0.004). This indicates that surgency moderated the effect of the intervention on Parent–Child Dysfunctional Interactions (see Table 3). For families in the intervention group, higher values of surgency were associated with lower scores on Parent–Child Dysfunctional Interaction scores. The linear slope of the relationship between surgency and Parent–Child Dysfunctional Interaction, adjusting for other covariates, was −2.80 for the intervention group and 0.61 for the control group. Each one-unit increase in surgency was associated with a 2.80-unit reduction in Parent–Child Dysfunctional Interaction for the intervention group.

For the Difficult Child subscale of the PSI, the interactions of effortful control and group (*p* = 0.21) and negative affect and group (*p* = 0.21) were not significant. For the Parental Distress subscale of the PSI, models testing the interaction between group and effortful control (*p* = 0.30), negative affect (*p* = 0.28), and surgency (*p* = 0.75) were not significant.

### Family Functioning

We ran three models—one with each subscale of the EBCQ VS/IBQ-R VS as a moderator—for the FAD General Functioning scale. None of the models showed a statistically significant interaction between temperament and group. Graphics showing a visual depiction of the interactions we tested are in the Supplemental Materials.

### Parent–Child Interactions

For each of the two composite scores for the Three Bags Task, we ran three models—one model with each subscale of the ECBQ VS/IBQ-R VS as a moderator—for a total of six models. Graphics showing a visual depiction of the interactions are in the Supplemental Materials. For the Positive Engagement composite score, models testing the interaction between group and effortful control (*p* = 0.58), negative affect (*p* = 0.66), and surgency (*p* = 0.35) did not show significant moderation. For the Sensitivity composite score, models testing the interaction between group and effortful control (*p* = 0.71) and surgency (*p* = 0.53) did not show significant moderation. The overall model testing the interaction between negative affect and group was significant (LRT: *χ*^2^_(df = 9)_ = 122, *p*-value < 0.001). There was a significant interaction between negative affect and group membership (*F*_(1, 65.4)_ = 5.37, *p* = 0.02). This indicates that negative affect moderated the effect of PFR on Sensitivity. Table 3 shows the full results for this model. The linear slope of the relationship between negative affect and Sensitivity, adjusting for covariates, was 0.06 for the intervention group and -0.19 for the control group. This means that for intervention families, higher values of negative affect were associated with higher Sensitivity; each 1-unit increase in negative affect was associated with a 0.06-unit increase in Sensitivity.

## Discussion

Evidence-based parent–child interaction interventions are increasingly being tested in field settings, showing promising results with diverse, low-income samples. This exploratory study examined the role played by a child characteristic—temperament—in influencing the effectiveness of the PFR intervention as an EHS enhancement delivered by home visiting staff. Though prior randomized trials (Oxford et al., 2016; Spieker et al., 2012) show that PFR produces positive impacts in parenting, including increased sensitivity, we did not find direct effects of PFR on the outcomes examined in the current study. However, we hypothesized that temperament would moderate the effectiveness of PFR on parent and family outcomes, and this was partially supported. Our findings are consistent with models of differential susceptibility (e.g., Belsky & Pluess, 2009; Kiff et al., 2011), which argue that children’s reactions and responses to positive or negative experiences with their environments (including parenting interactions) differ based on inherent child characteristics. In some families, child temperament appears to be an important characteristic when examining PFR impacts in an EHS sample. Indeed, our results reflect PFR priorities of being responsive in caregiver-child interactions, being attuned to young children’s needs, and acknowledging children’s unique characteristics.

Specifically, our results show that surgency moderated the effectiveness of PFR on the Difficult Child and Parent–Child Dysfunctional Interaction subscales. In both cases, in families with more extraverted children, parents reported lower levels of parenting stress after completing PFR. Thus, while not found for the entire PFR group, reductions in parenting stress were present when children demonstrated higher levels of surgency. For our measure of parent–child interaction, child temperament also played a moderating role. However, for this measure, the component of child temperament found to be most important was negative affect. When children had higher levels of negative affect, parents showed more sensitivity in interacting with their children after completing PFR. While the body of research examining differential effectiveness of early childhood parenting interventions is small, our finding that child temperament moderates parenting outcomes helps build upon prior findings. Previous studies looking into the dimensions of temperament have not yet identified consistent predictions of whether families of children with more or less difficult temperament characteristics benefit most from positive parenting (e.g., Kochanska et al., 2007; Slagt et al., 2016). However, one relevant study from Dalimonte-Merckling and Brophy-Herb (2019) used a national EHS sample and employed the same measures of parenting stress and parent–child interaction as the current study. These researchers conducted a latent profile analysis where two of the three identified profiles (representing 428 of 2,504 families) were characterized by mother–child dyads with temperamentally reactive children and mothers having challenges with parent–child interactions and parenting stress. The smallest, but most worrisome, of these groups (55 families) contained children who were highly emotionally reactive paired with mothers experiencing high parenting stress as well as negative interactions with their children. These results, along with our findings, suggest that interventions such as PFR may result in different kinds of benefits for families, when children demonstrate high levels of surgency as well as when they demonstrate high levels of negative affect.

A limitation of our study involves the use of parent-report data to assess parenting stress, family functioning, and child temperament. Mothers may have answered based on current mental states at the time data were collected. Though we identified child temperament as the focus of our moderated analysis, it may also reflect differences in caregiver temperament as well as other experiences, including the child’s prenatal experiences. Also related to child temperament, while surgency moderated PFR effectiveness for Difficult Child and Parent–Child Dysfunctional Interaction subscales of the PSI, scores on those subscales were themselves highly correlated. This may contribute to the similar moderated effects that we observed. Other limitations include the relatively small sample size and higher attrition rate in the PFR condition than the control condition (56% vs. 72% retained at follow-up), likely due to PFR participation placing more demands on families. This suggests some self-selection of families remaining in PFR. It was necessary to exclude additional families from our dataset due to incomplete temperament data. Main effect findings similar to those in prior PFR studies with higher-risk samples (Oxford et al., 2016; Spieker et al., 2012) could potentially be observed using a larger EHS sample affording more statistical power. Also, families in our control group received ongoing EHS services from PFR-trained staff who were using the intervention with other families due to our waitlist-control design. Finally, a relatively small number (3 of 18) of the tested interactions were statistically significant, and we did not adjust *p*-values for Type 1 error rates across the models, which increases the possibility that some results may arise as the result of chance. While the findings are suggestive of positive impacts of PFR as moderated by child temperament, this is another reason to view the findings as preliminary until investigated by further research.

It is important to emphasize that although families in our control group did not receive PFR, they were participating in both EHS and Parents as Teachers, which are individually designated by the U.S. Department of Health and Human Services, Administration for Children and Families (n.d.) as models with sufficient evidence of effectiveness to be used in federally funded home visiting projects. Due to our study design, including our focus on the additive effect of a supplemental intervention as well as the use of PFR-trained home visitors or family service workers with families in the control group, these results likely underestimate potential impacts of PFR that would be found in a study using a true no-treatment comparison group. This may offer an additional explanation of why we did not find main effects of the intervention. The finding that differences in child temperament were linked with positive parent outcomes when families participated in PFR highlights the potential of PFR to add value to EHS services that already emphasize parent–child relationships through use of curricula such as PAT. A key contribution of the current study is the test of PFR effectiveness under real-world EHS conditions.

An additional priority of our demonstration of PFR in everyday conditions involved training existing EHS staff to deliver a parent–child interaction intervention to families on their current caseloads, to enhance ongoing services, build upon existing relationships, and promote sustainability. We selected PFR because its training and professional development requirements were accessible to staff employed by our partner EHS agency. Potentially, if home visiting staff were hired based on their reflective capacity or other skills emphasized in PFR, this would allow for greater accuracy in estimating PFR impacts. This was not feasible during our time-limited study but would be more feasible in EHS programs involved in long-term implementation of an intervention. In addition to examining family outcomes, we interviewed home visitors employing PFR. They identified numerous strengths of the intervention, placed a high value on PFR content, and said it helped improve their home visiting practice (Han et al., 2016). Another consideration would involve hiring specialized staff focused only on intervention delivery as a supplemental service, rather than integrating PFR as an enhancement in home visits. While this expert approach can be effective (Berlin et al., 2018), we were concerned it would not be sustained once our study concluded. Our model for PFR delivery by EHS home visiting staff ultimately was sustainable, as the partnering agency continues to offer PFR.

Our results suggest that agencies selecting enhancements to home visitation models should consider both characteristics of children and families and their own organizational capacity to implement the intervention. The match between a parent–child interaction intervention with families’ needs and characteristics may result in differences in whether the intervention can be fully utilized and in which families might derive the greatest benefits. Although further research is needed, in this study PFR appeared to be especially beneficial for EHS families with children showing high levels of surgency or negative affect.

## Supplementary Information

Below is the link to the electronic supplementary material.

Supplementary file1 (PDF 1002 kb)
