# Supplementary material for: Child Temperament as a Moderator of Promoting First Relationships Intervention Effects Among Families in Early Head Start
Source: Prev Sci. 2022 Jan 21;26(Suppl 1):41–51. doi: 10.1007/s11121-022-01340-0 (PMC12053191; doi:10.1007/s11121-022-01340-0)

Supplemental Materials

CONSORT Flow Diagram

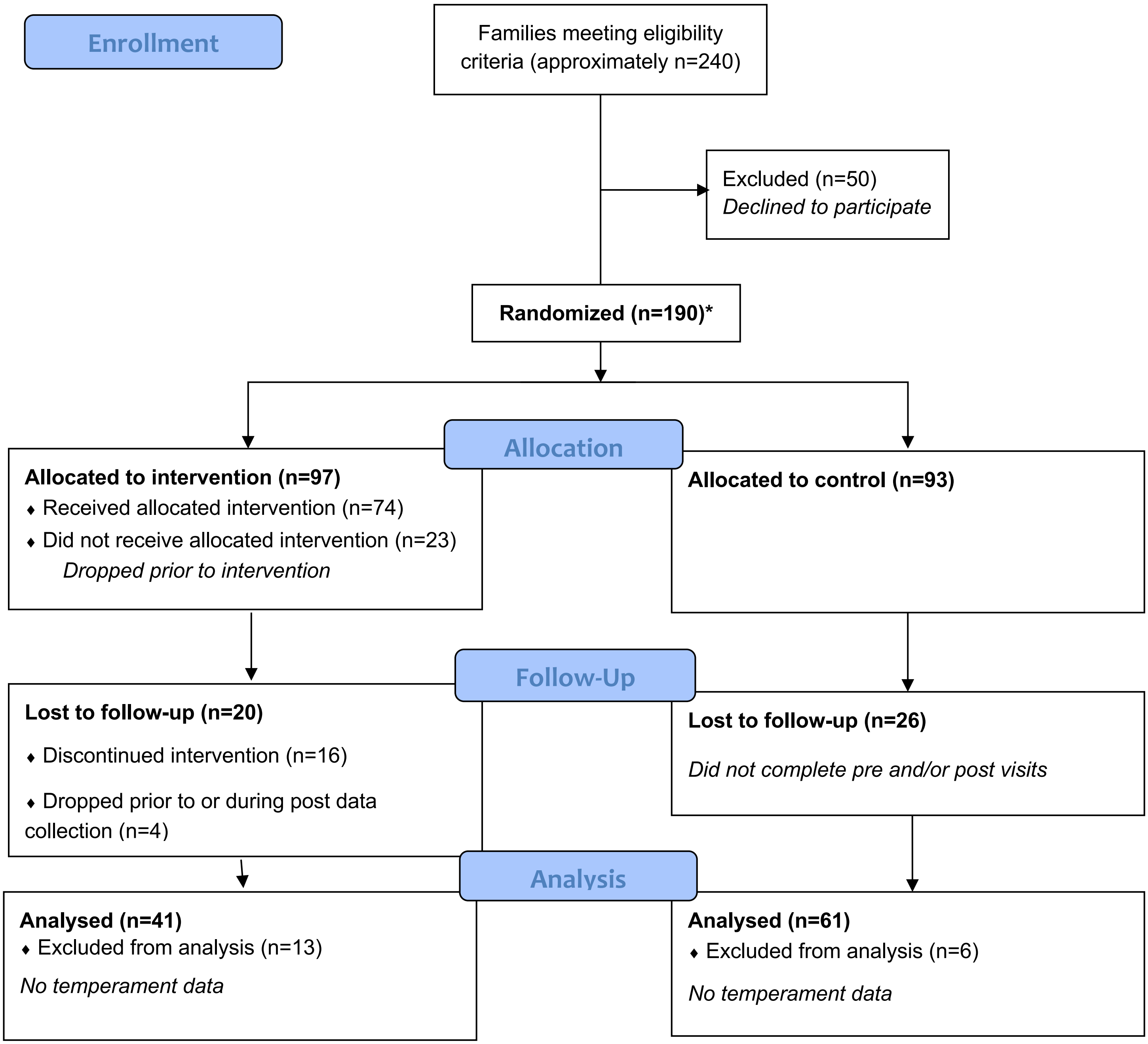

*Note: 4 of the 190 families were purposefully selected for the intervention group rather than randomized. This was done at the start of intervention implementation when the first group of home visitors had reached fidelity on PFR.

Pearson Correlations of Outcome Variables and Temperament Constructs

Variables	1	2	3	4	5	6	7	8	9	10
1. PSI Total Score	_									
2. PSI Parental Distress	.885**	_								
3. PSI Parent-Child Dysfunctional Interaction	.868**	.66**	_							
4. PSI Difficult Child	.89**	.64**	.71**	_						
5. FAD General Functioning	.58**	.51**	.47**	.44**	_					
6. Three Bags Positive Engagement	.13	.20*	-.01	.08	.01	_				
7. Three Bags Sensitivity	.00	.09	-.00	-.08	-.06	.52**	_			
8. Effortful control	-.47**	-.30**	-.45**	-.51**	-.23*	.00	.13	_		
9. Negative Affect	.21	.13	.10	.29**	.32**	.11	-.20	.01	_	
10. Surgency	-.30**	-.22*	-.401**	-.24*	-.18	.32**	.19	.36**	.26*	_

* $p < .05$, ** $p < .01$

Figures Showing Simple Slopes with Interaction Effects for Each Outcome Variable/Temperament Subscale Combination

PSI Difficult Child with Effortful Control Interaction

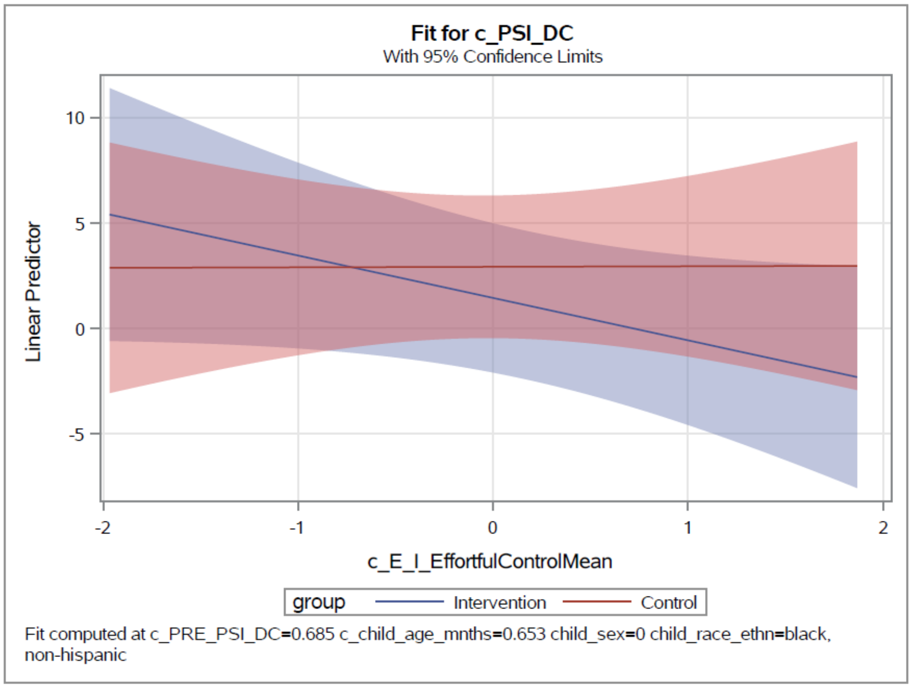

PSI Difficult Child with Negative Affect Interaction

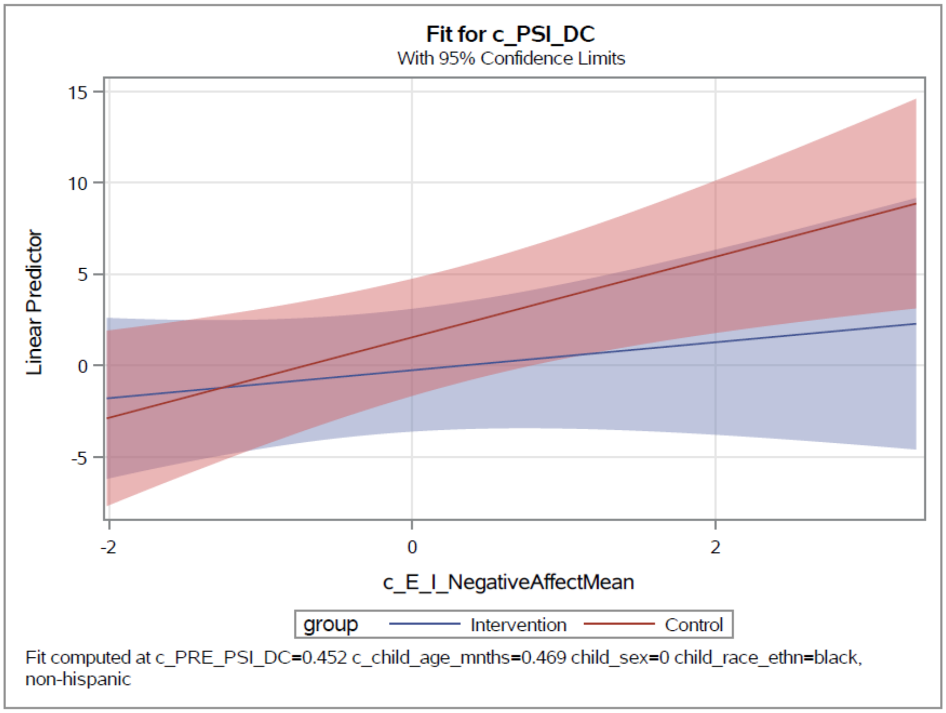

PSI Difficult Child with Surgency Interaction

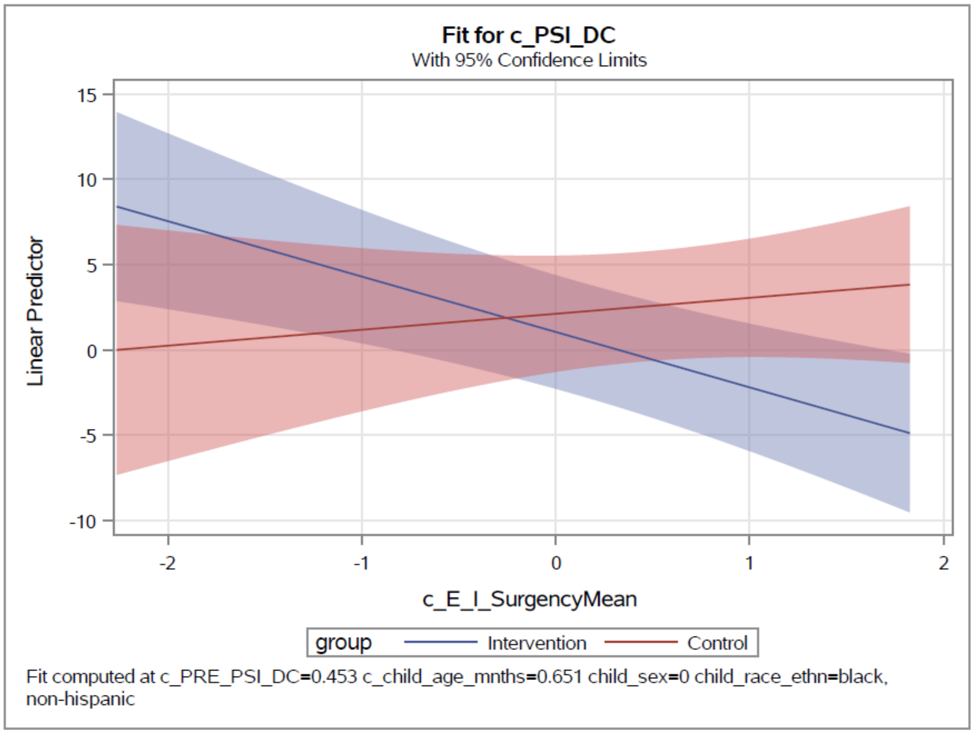

PSI Parental Distress with Effortful Control Interaction

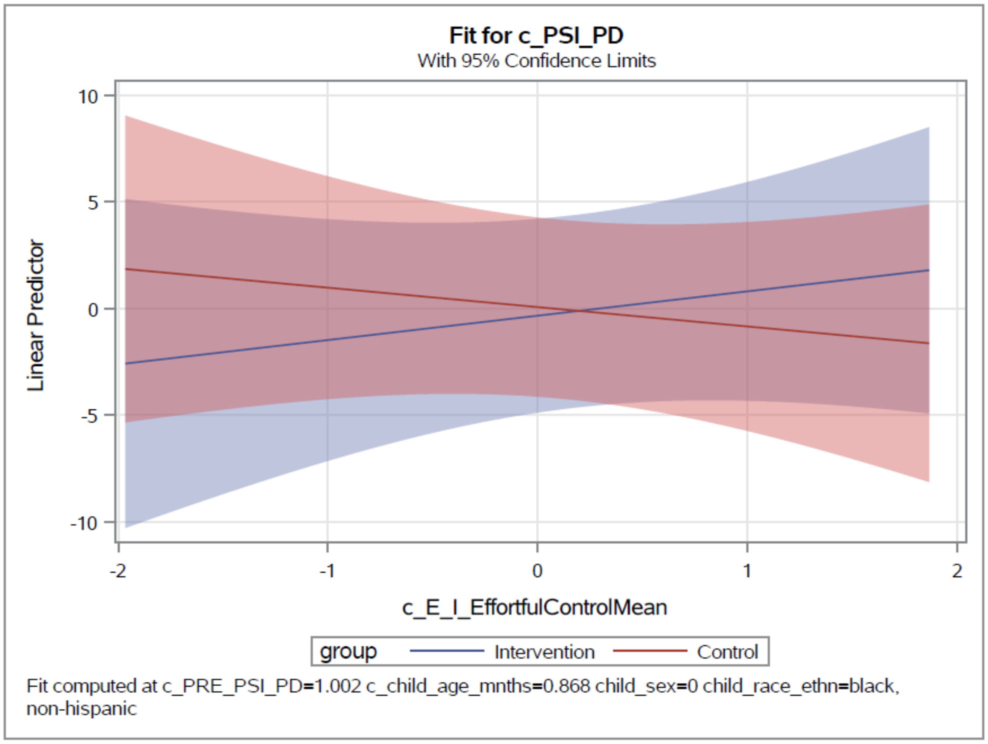

PSI Parental Distress with Negative Affect Interaction

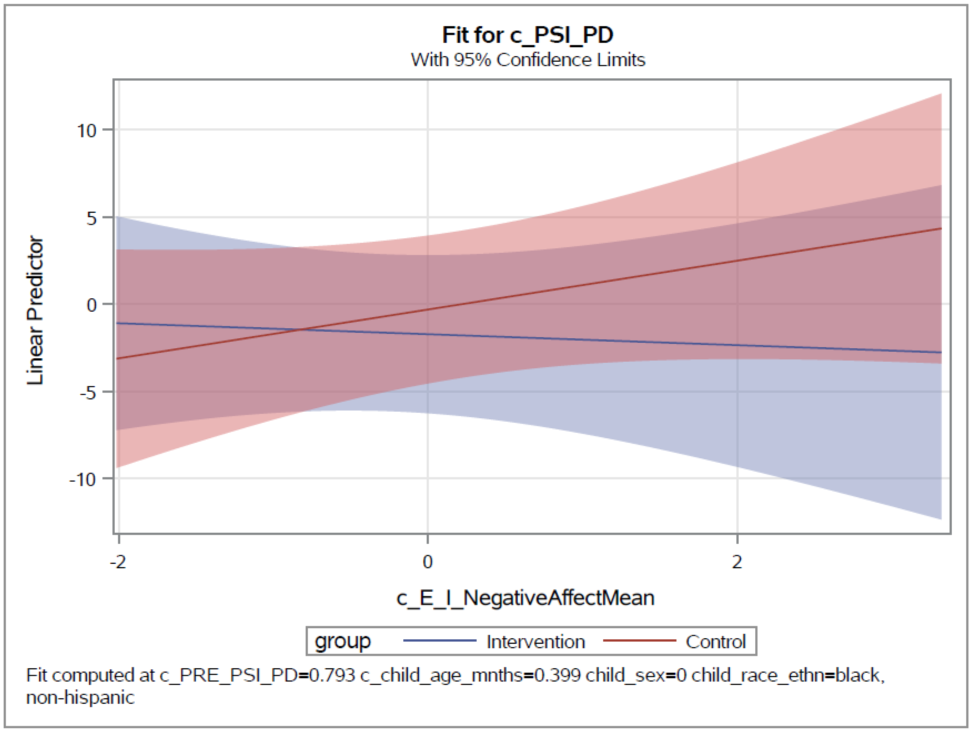

PSI Parental Distress Interaction with Surgency Interaction

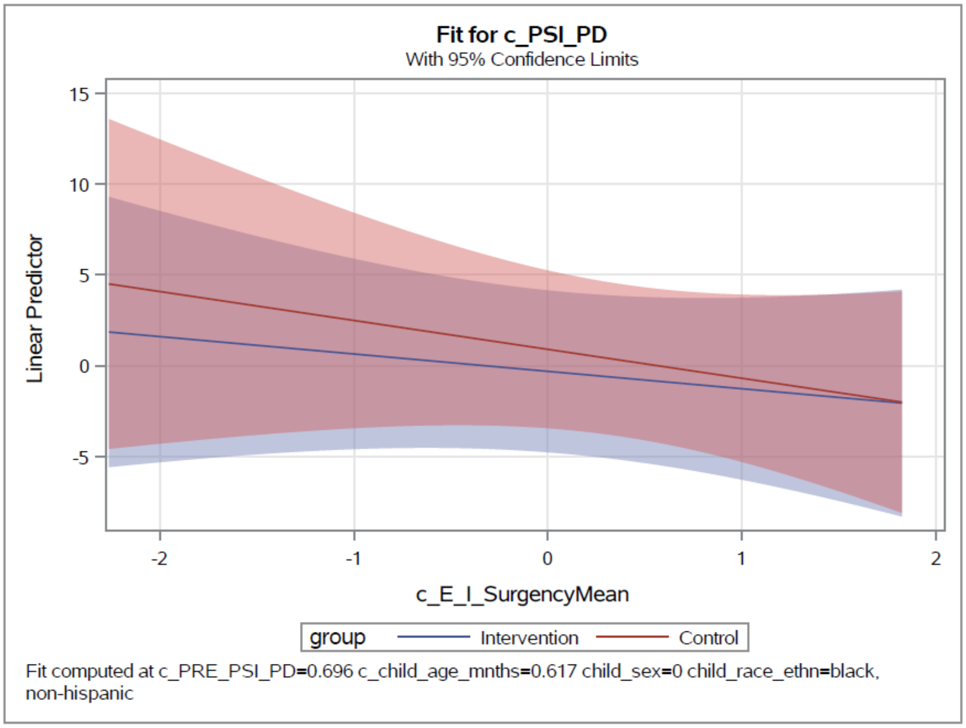

PSI Parent-Child Dysfunctional Interaction with Effortful Control Interaction

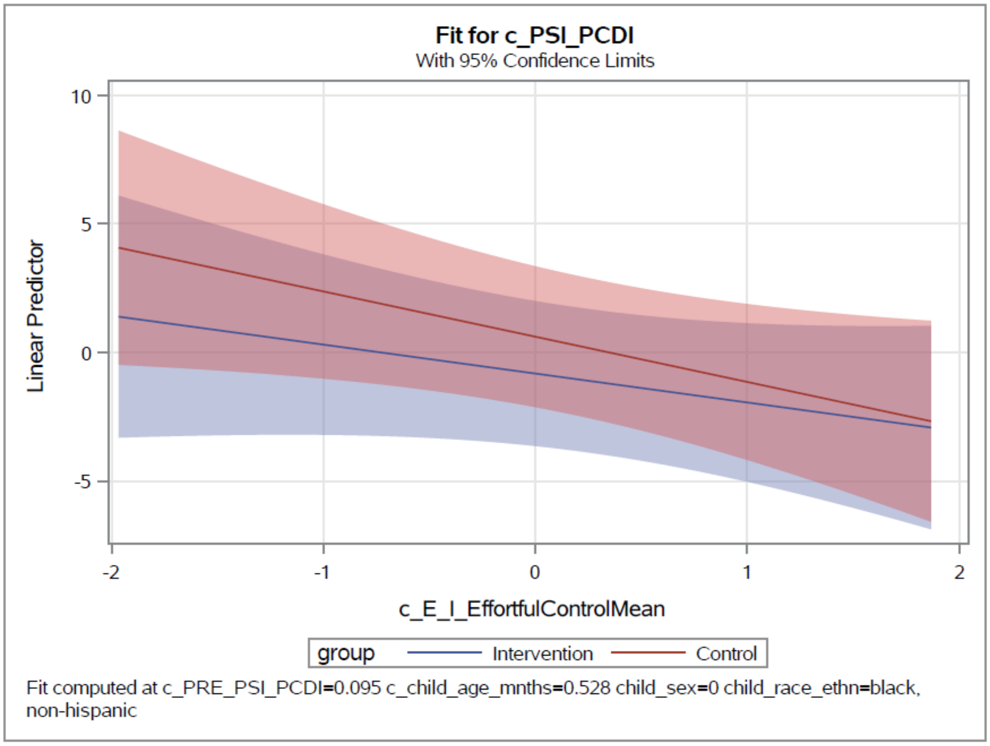

PSI Parent-Child Dysfunctional Interaction with Negative Affect Interaction

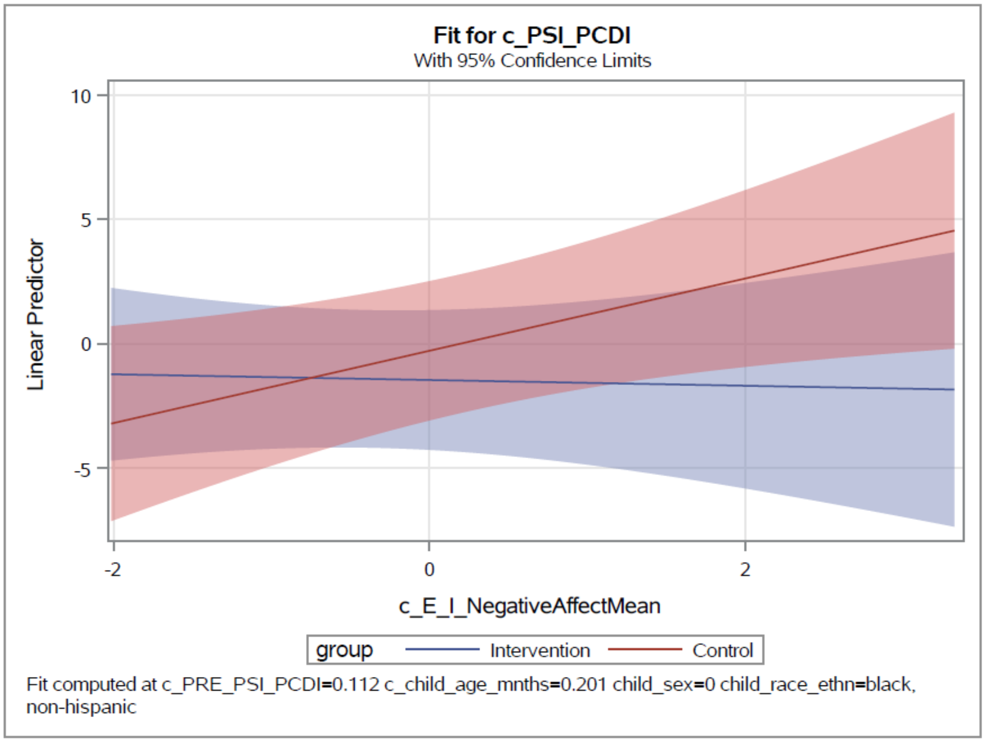

PSI Parent-Child Dysfunctional Interaction with Surgency Interaction

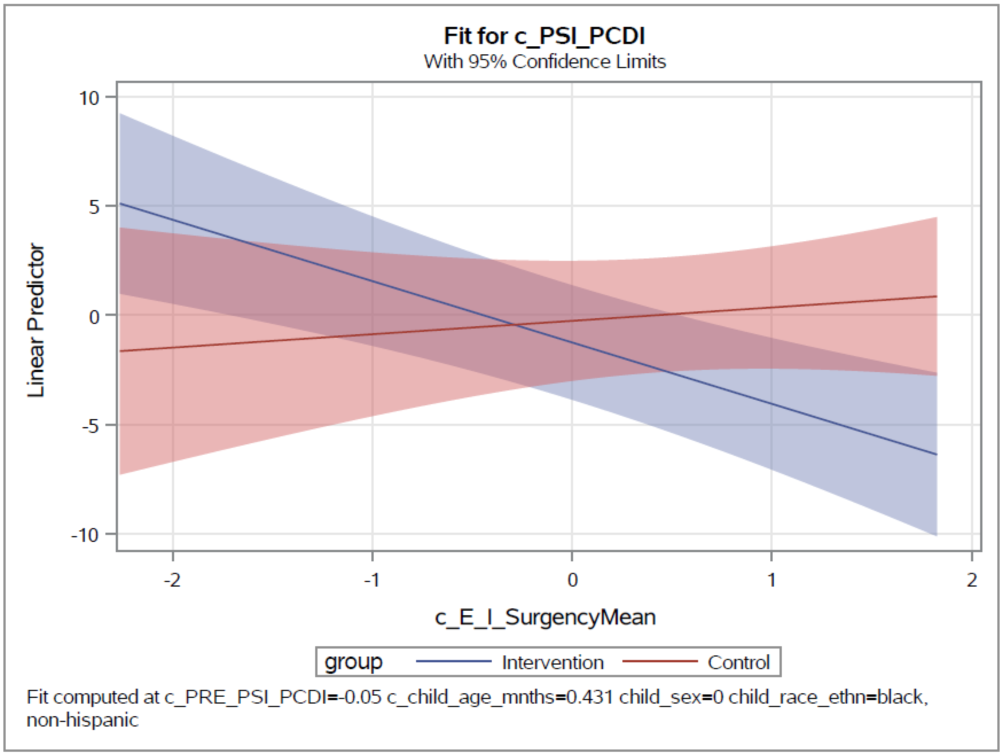

FAD General Family Functioning with Effortful Control Interaction

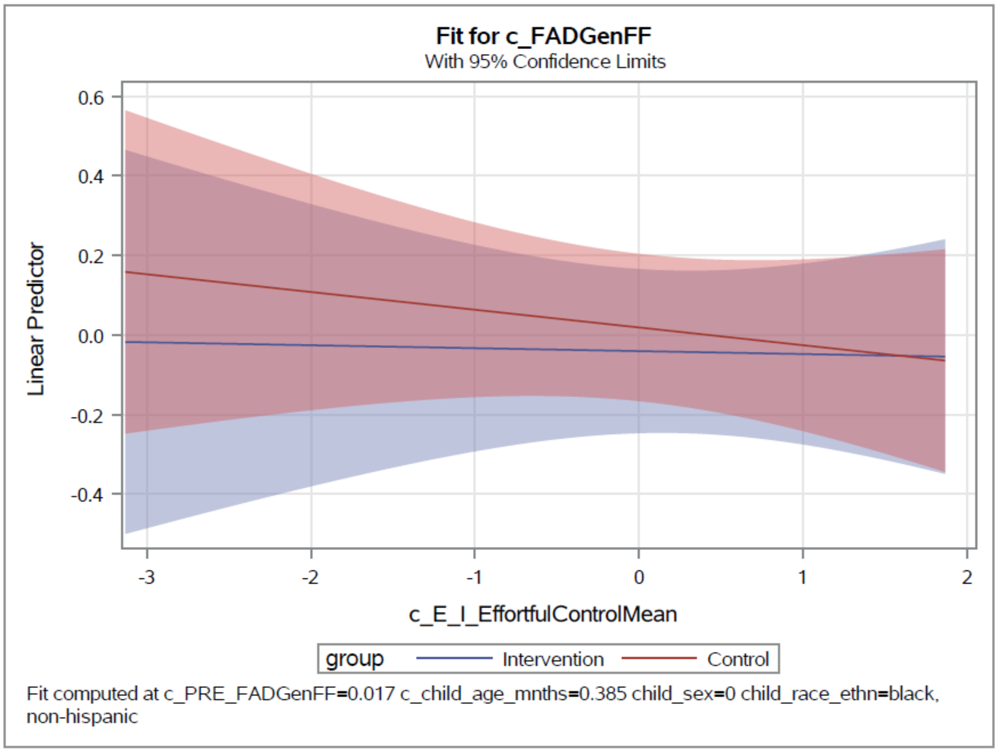

FAD General Family Functioning with Negative Affect Interaction

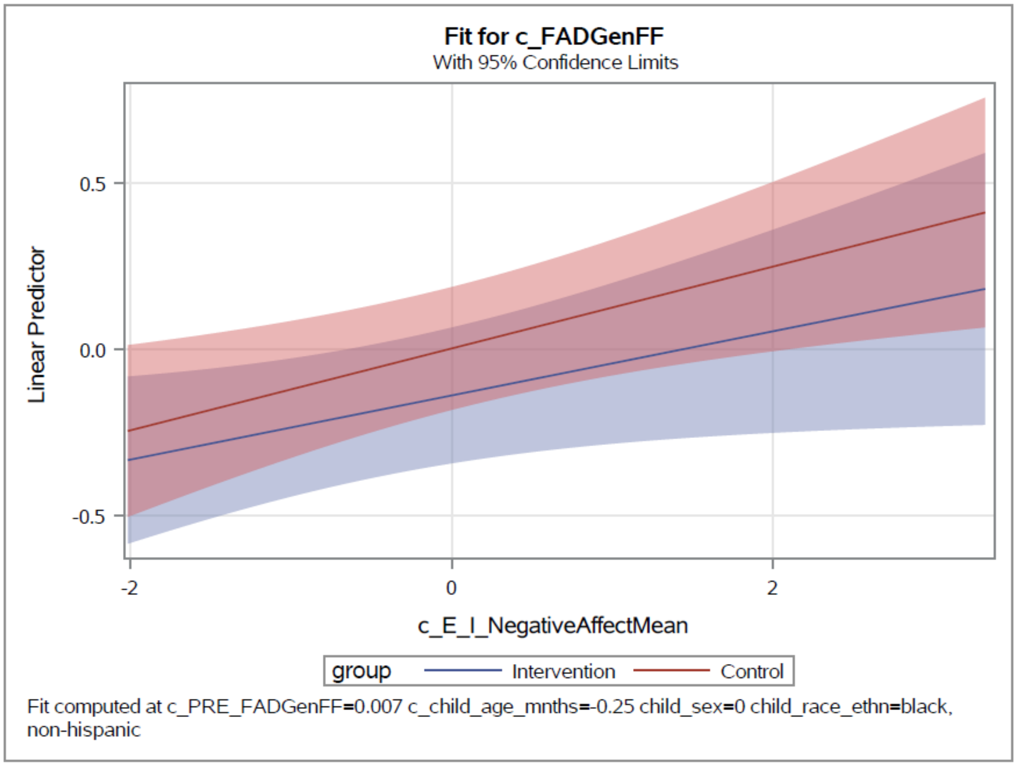

FAD General Family Functioning with Surgency Interaction

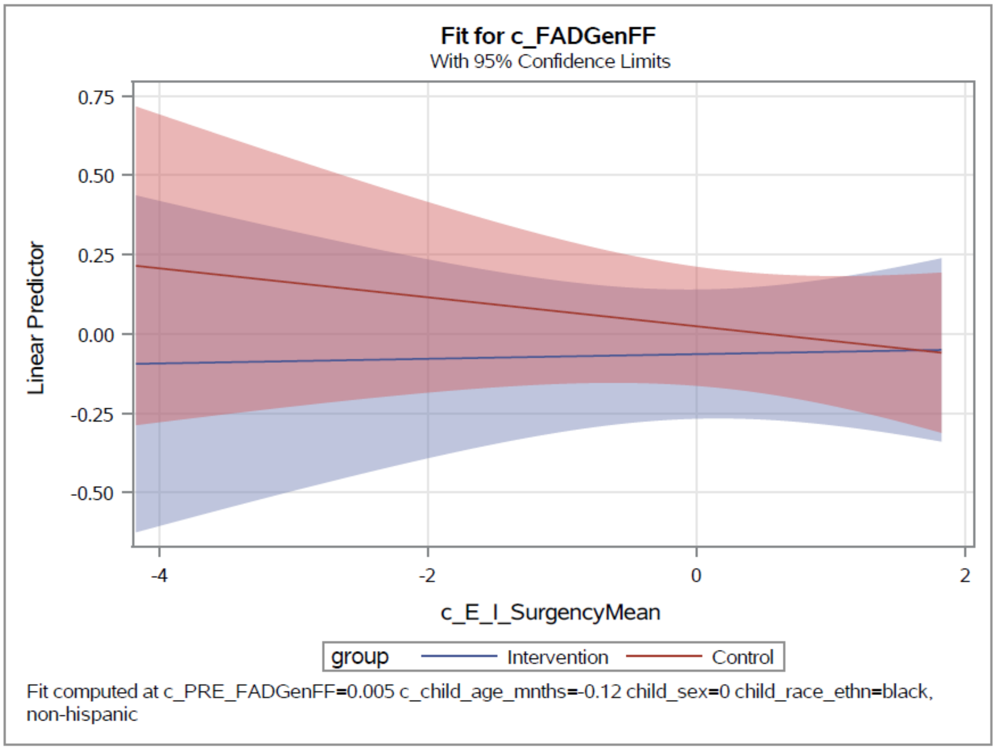

Three Bags Sensitivity with Effortful Control Interaction

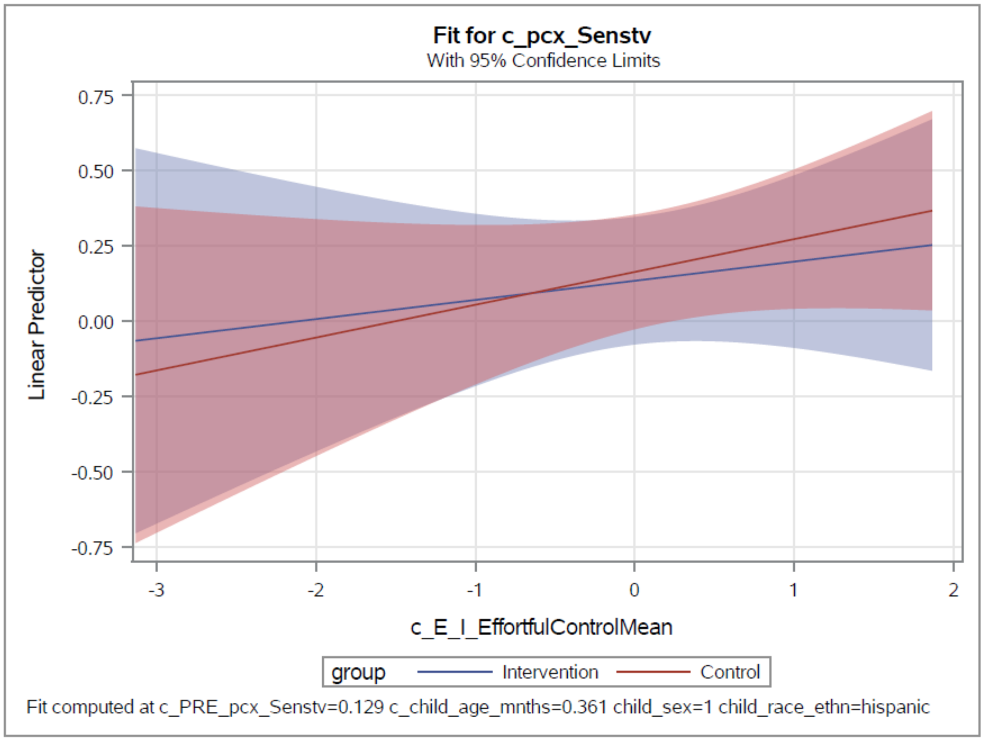

Three Bags Sensitivity with Negative Affect Interaction

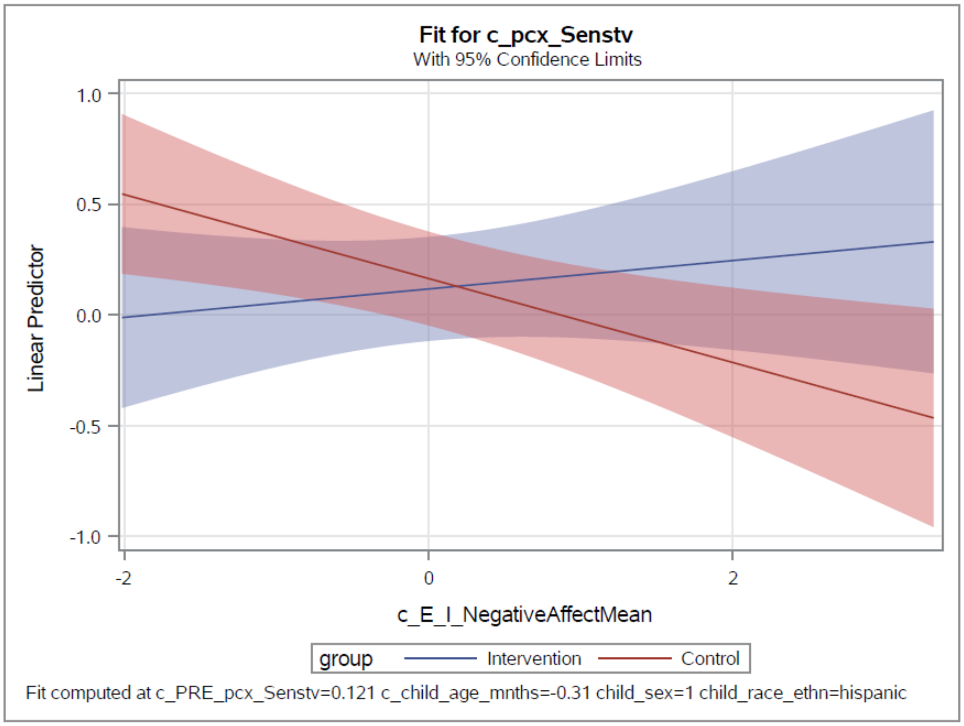

Three Bags Sensitivity with Surgency Interaction

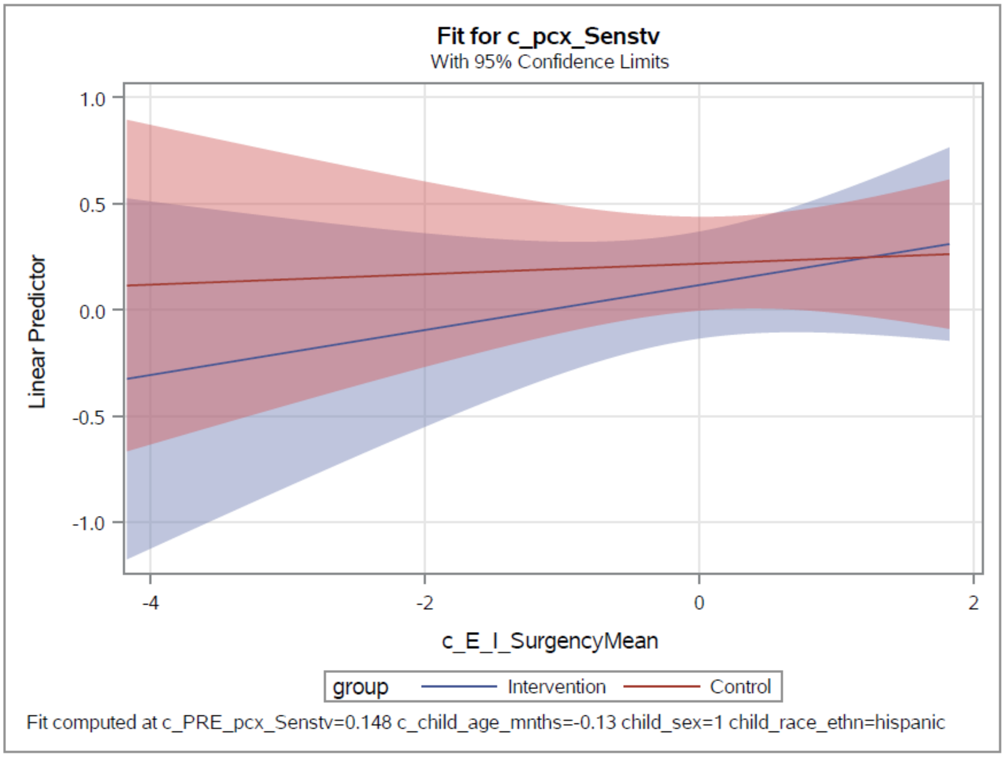

Three Bags Positive Engagement with Effortful Control Interaction

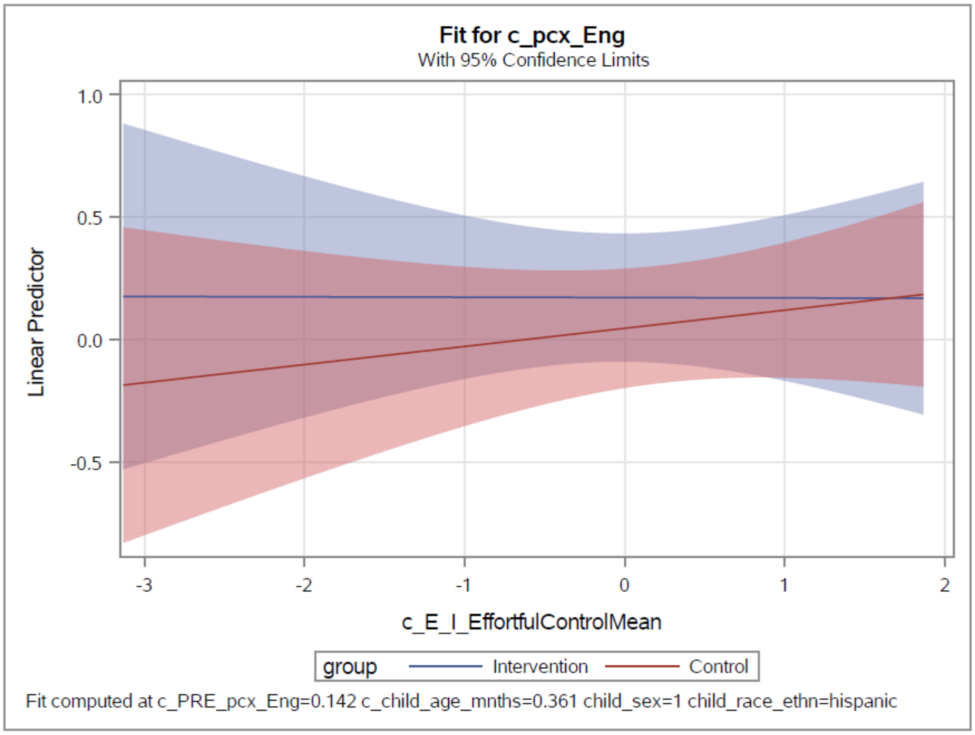

Three Bags Positive Engagement with Negative Affect Interaction

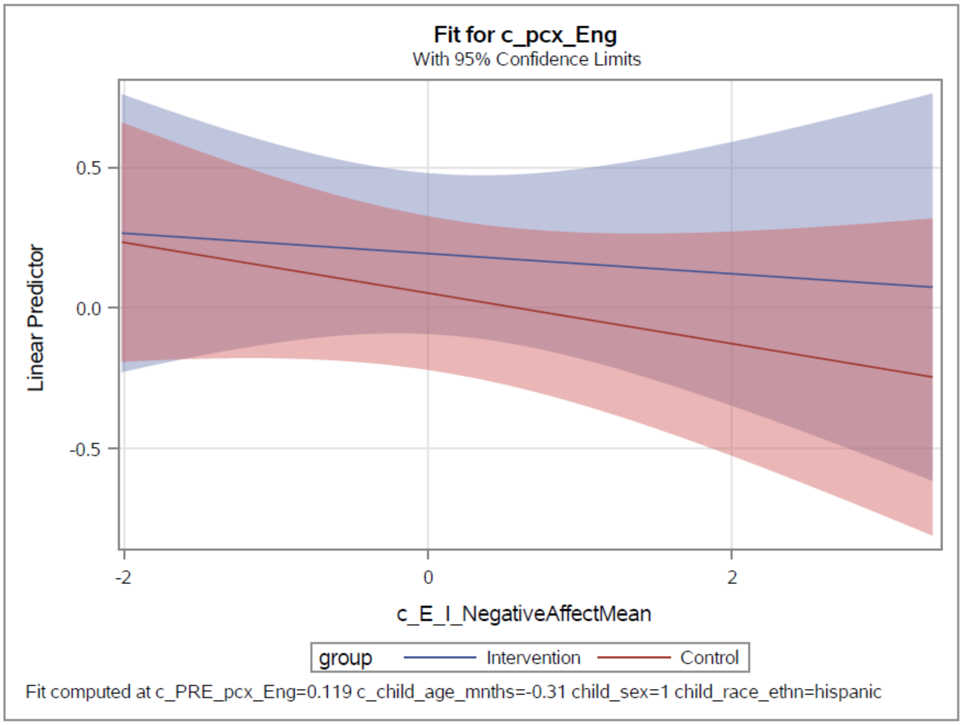

Three Bags Positive Engagement with Surgency Interaction

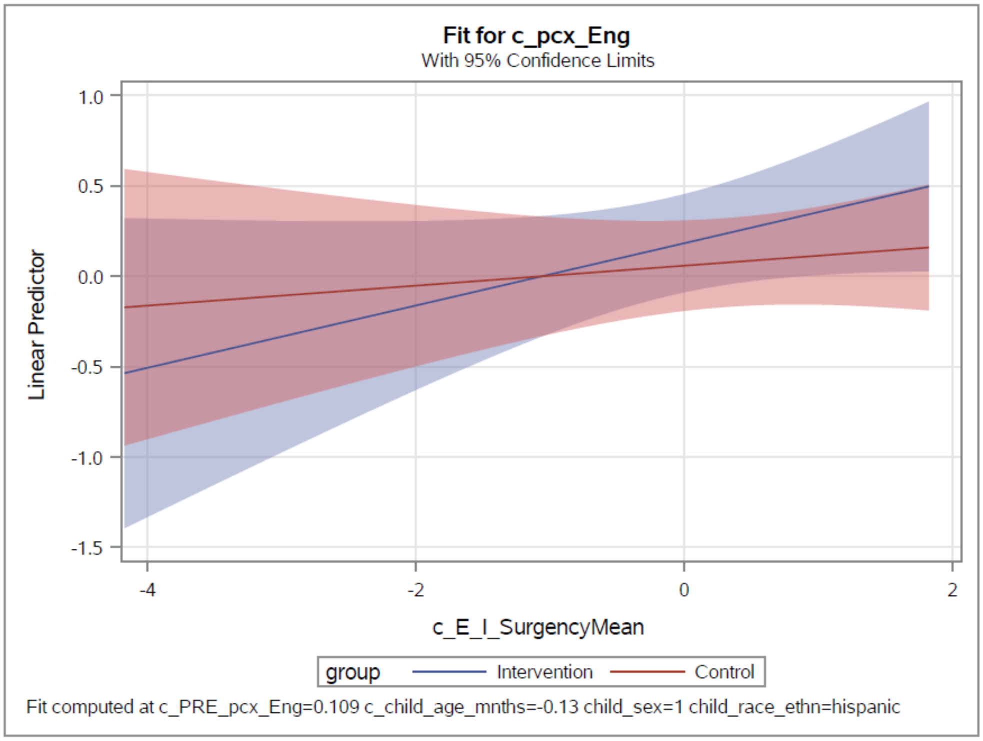

Supplement: Supplementary file 1 — Supplementary file1 (PDF 1002 kb) [file 11121_2022_1340_MOESM1_ESM.pdf]
